# Modulation of Working Memory and Resting-State fMRI by tDCS of the Right Frontoparietal Network

**DOI:** 10.1155/2021/5594305

**Published:** 2021-07-26

**Authors:** Monika Pupíková, Patrik Šimko, Martin Gajdoš, Irena Rektorová

**Affiliations:** ^1^Central European Institute of Technology-CEITEC, Masaryk University, Applied Neuroscience Research Group, Brno, Czech Republic; ^2^Faculty of Medicine, Masaryk University, Brno, Czech Republic; ^3^Central European Institute of Technology-CEITEC, Masaryk University, Multimodal and Functional Neuroimaging Research Group, Brno, Czech Republic; ^4^First Department of Neurology, Faculty of Medicine, Masaryk University and St. Anne's University Hospital, Brno, Czech Republic

## Abstract

Many cognitive functions, including working memory, are processed within large-scale brain networks. We targeted the right frontoparietal network (FPN) with one session of transcranial direct current stimulation (tDCS) in an attempt to modulate the cognitive speed of a visual working memory task (WMT) in 27 young healthy subjects using a double-blind crossover design. We further explored the neural underpinnings of induced changes by performing resting-state fMRI prior to and immediately after each stimulation session with the main focus on the interaction between a task-positive FPN and a task-negative default mode network (DMN). Twenty minutes of 2 mA anodal tDCS was superior to sham stimulation in terms of cognitive speed manipulation of a subtask with processing of objects and tools in unconventional views (i.e., the higher cognitive load subtask of the offline WMT). This result was linked to the magnitude of resting-state functional connectivity decreases between the stimulated FPN seed and DMN seeds. We provide the first evidence for the action reappraisal mechanism of object and tool processing. Modulation of cognitive speed of the task by tDCS was reflected by FPN-DMN cross-talk changes.

## 1. Introduction

The integrity and connectivity of large-scale brain networks were shown to play a crucial role in the individual performances of distinct cognitive tasks—namely, in the interplay between the task-positive frontoparietal network (FPN) and the “task-negative” default mode network (DMN) [1–3]. In accordance with this notion, we showed in young healthy subjects (HY) that manipulation of the excitability of the posterior parietal cortex (that is part of the FPN) with repetitive transcranial magnetic stimulation (rTMS) led to remote cognitive task-induced BOLD signal decreases of the DMN along with enhanced cognitive speed [4]. While the frontoparietal cortices contribute to adaptive behavior control via the flexible encoding of task demands and desired outcomes and the top-down modulation of processing in other brain regions [5], the DMN is involved in mind wandering and various aspects of self-referential internally directed processing, and its activity is suppressed by most goal-directed tasks [2, 6]. Disruption to both the FPN and the DMN is a common finding in Alzheimer's disease and Parkinson's disease, i.e., the most common neurodegenerative brain diseases leading to cognitive decline in aging populations [7–10]. Some authors have suggested that the magnitude and direction of task-related hemodynamic responses within regions engaged in the task-positive and task-negative networks are related to the intrinsic connectivity patterns between these networks [11], and it may therefore be plausible to use resting-state functional connectivity (rs-FC) measures between the major task-positive and task-negative brain networks to examine the neural correlates of cognitive modulation induced by targeted noninvasive brain stimulation.

Among various noninvasive stimulation techniques, transcranial direct current stimulation (tDCS) was proposed as an inexpensive and easily administered method for experimental and potentially also for clinical use in an attempt to modulate brain function [12]. The tDCS causes only very mild side effects such as itching and tingling [13], and unlike rTMS, it seems optimal even for supervised home care [14]. tDCS applies a weak direct electric current through two electrodes placed over the scalp with the goal of modulating underlying cortical excitability [15]. The effects of tDCS lead to subthreshold changes in membrane potentials towards depolarization/hyperpolarization [16]. The tDCS efficacy for modulating cognitive accuracy and speed is still in question [17–19]. Moreover, little is known about the neural underpinnings of tDCS-induced cognitive changes in the healthy brain.

In the current study, we used tDCS with the aim of modulating visual working memory (WM) performance. WM refers to the limited capacity system for online temporary storage and simultaneous manipulation of information to be employed in ongoing processing [20, 21]. It is related to higher-order cognitive skills such as multitasking or learning [22] therefore central to the execution of a variety of daily functions. One influential way how to conceptualize working memory is a multicomponent system comprising modality-specific temporary memory systems, which store information and refresh memory traces, and a supervisory system (so-called central executive) that is tasked with various executive functions such as focusing and switching attention, coordinating the storage systems as well as activating the contents within long-term memory [23]. The loss of WM efficiency related to either aging or a pathological process is hypothesized to have various neurobiological roots, among which altered functioning of the FPN plays an important role [24–26]. We used a right-sided frontoparietal montage based on the results of our previous visual WMT fMRI research (matching objects from conventional vs. unconventional views) performed in patient groups with mild cognitive impairment due to a neurodegenerative brain disease [27]. The patients displayed cognitive deficits along with deficient involvement of the right-sided multimodal (domain general) frontoparietal regions, suggesting altered top-down processing; the activation of cortical areas within the ventral and dorsal visual pathways (i.e., bottom-up processing) remained preserved as compared to controls [27]. We hypothesized that tDCS of the right frontoparietal regions would alter the efficiency and cognitive speed of the same visual WMT by modulating the top-down control of visual processing, even in healthy young individuals. We further hypothesized that the tDCS-induced cognitive speed modulations of the offline WMT would be reflected by intervention-induced changes in the intrinsic functional connectivity between the FPN and the DMN.

## 2. Materials and Methods

### 2.1. Subjects

Participants were recruited from a university environment (we targeted mainly students or early career researchers) in the age range between 18 and 35 years. Participants did not receive any monetary compensation. Exclusion criteria were the presence of any psychiatric or neurological disorder, cognitive deficit based on the Montreal Cognitive Assessment (cut-off score 26 points), and drug or alcohol abuse. All subjects had normal or corrected-to-normal visual acuity. Written informed consent was obtained from all subjects prior to the experiment. The study was performed according to the guidelines of the Declaration of Helsinki and approved by the local ethics committee. The study was registered at clinicaltrials.gov with reference ID NCT04134195.

### 2.2. Study Protocol

**Figure 1:**
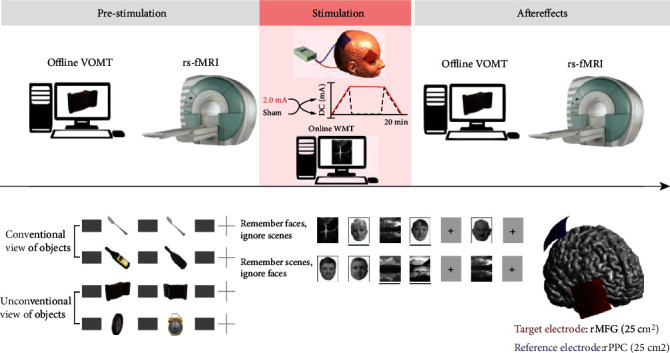
Experimental design and methods. (a) The crossover design involved two sessions with real 2 mA stimulation/sham tDCS with concurrent working memory task. Prior to and after the stimulation, participants performed a visual object matching task and underwent resting-state fMRI. (b) Offline VOMT—subjects respond whether the two consecutive objects are the same or different by pressing the YES/NO button in two difficulty levels (conventional view of objects—lower difficulty level; unconventional view of objects—higher difficulty level). (c) Online WMT—subjects view a block of faces and scenes (2 + 2, randomized order) preceded by a specific command on how to react to a probe which follows each block. Subjects respond whether the probe is consistent/inconsistent with the prior instruction by pressing the YES/NO button. (d) Montage for real and sham tDCS.

In this double-blind controlled trial with a crossover design, the subjects were tested in two different experimental sessions, each corresponding to the active or sham stimulation conditions (see Figure 1). Prior to the experimental sessions, each subject underwent T1 MRI sequence scanning to enable individual targeting of tDCS montage (for further details, see below) and practiced the experimental tasks in order to reduce the practice effects, which are most prominent between the first and the second sessions [28]. Each session lasted approximately 2 hours and included a visual object matching task (VOMT) [27] and a resting-state fMRI examination—both performed before and immediately after each tDCS session (active, sham). The tDCS was run outside the scanner simultaneously with another visual working memory task (online WMT) in order to enhance the stimulation aftereffects [29, 30], see Figure 1. The order of experimental conditions was counterbalanced across subjects.

### 2.3. Visual Working Memory Tasks

Throughout the study, we used two different visual working memory tasks. A visual-object matching task (VOMT) [27] was our main behavioral outcome task, and it was performed before and immediately after tDCS (i.e., the offline VOMT).

The task consists of multiple successive paired images of common objects. The second image of each pair is either the same or different from the first image (different object identity or object orientation). Participants are instructed to respond as quickly as possible by pressing a YES button if the second object of the paired images is the same as the first object (regardless of spatial orientation) or by pressing a NO button if the second object is different. Each trial comprised the following sequence: a mask stimulus (1 s), followed by the picture of the first object (1 s), followed by a mask (1 s), followed by the picture of the second object (1 s), followed by a mask (1 s), ending with a fixation cross (5 s). Conditions are presented in a randomized order (see Figure 1(b)). Overall, participants viewed 40 trials in total, 20 trials in each condition. The task takes 7 minutes to accomplish. We collected the number of correct responses and reaction times (RT) of both conditions—conventional view (lower difficulty level) condition and unconventional view (higher difficulty level) condition with rotated object views. We used different versions of the task for every session, balanced in difficulty. Based on previous tDCS study results in young participants [31, 32], we specifically focused on the unconventional view of object condition subtask of the VOMT (i.e., the subtask with a higher cognitive load) and on processing speed rather than task accuracy as our primary outcome measure.

The online WMT involved a working memory task with faces and outdoor scenes adopted from Gazzaley et al. [33] which was performed during the stimulation session (active, sham). The task consisted of two subtasks in which aspects of visual information were maintained constant while the target instruction changed. Participants were instructed to attend to one particular category and ignore irrelevant distractor category, so there were two kinds of instructions: (1) “remember faces and ignore scenes” vs. (2) “remember scenes and ignore faces.” During each trial, participants viewed a series of four images—two faces and two scenes—in a randomized order. Images were presented sequentially for 800 msec. After a retention period of 9 seconds, a probe was presented (consistent with the prior instruction) and participants responded whether or not the probe was present in the foregoing series. The task was performed for the whole duration of the stimulation, thus 20 minutes. In total, 48 trials were presented, and conditions 1 and 2 were randomized into 4 blocks consisting of 10 trials balanced in condition (see Figure 1(c)). The number of correct responses and reaction times (RT) was collected.

### 2.4. tDCS Stimulation Parameters

tDCS was performed through a battery-driven stimulator (DC-Stimulator Plus, neuroConn GmbH, Germany). The anode was positioned over the right middle frontal gyrus (rMFG; MNI = 44 40 -10) with the cathode over the right posterior parietal cortex (rPPC, MNI = 30 -55 52). We used the T1 MRI scan-based frameless stereotactic neuronavigation targeting with Brainsight 2, to specify the exact location of the electrode center in each individual. With this electrode montage, we aimed to modulate the excitability of the right frontoparietal multimodal regions based on a cortical parcellation atlas described in Yeo et al. [34]. The current of 2 mA was delivered using two rubber electrodes (5 × 5 cm) for 20 minutes, with initial ramp-up and final ramp-down phases. The electrode was held in place by conductive paste (Ten20 Conductive Paste gel, Weaver and Company). The sham stimulation was applied with the same settings, but the stimulator was turned off after 30 seconds. The impedance was controlled by the device throughout the session limited by the voltage at 26 V, not exceeding 15 k*Ω*. An excess of limits would have led to an automatic termination of stimulation.

### 2.5. Magnetic Resonance Imaging

MRI data were acquired with a 3.0 T Magnetom Siemens Prisma. We acquired T1 MPRAGE sequence (TR 1570 ms; TE 2.45 ms; voxel size 1 × 1 × 1 mm; FoV 256 × 226 mm; flip angle 8°; 160 transversal slices) in all participants to navigate the stimulation (as described above). A subgroup of 22 participants underwent resting-state fMRI (rs-fMRI; *n* = 22; TR 850 ms; TE 35.2 ms; voxel size 2 × 2 × 2 mm; FoV 208 mm; flip angle 45°; 80 transversal slices; 700 scans; multiband factor 8) prior to and immediately after each tDCS condition.

### 2.6. Data Analysis

#### 2.6.1. Analysis of Behavioral Data

We recorded the number of correct responses and reaction times (RT) in both tasks (see in Results). Based on the results of previous studies in young participants, including our own research [4, 35, 36], we predicted ceiling effects for task accuracy; we therefore focused on RT changes. Normality of data was assessed with Kolmogorov-Smirnov tests. Paired *t*-tests were used to compare differences (RT post–RT baseline) in VOMT performance between stimulation conditions (active vs. sham). The baseline RT in VOMT was measured before each sham or active stimulation session and calculated as their mean. The slopes of the reaction time learning curves (*β* RT) for the online WMT were compared between active and sham groups using paired *t*-tests. We further correlated online and offline effects by comparing *Δ* RT of VOMT and *β* RT of the online WMT. Data were corrected for learning effects during sessions and analyzed with SPSS 24.0 software.

#### 2.6.2. Analysis of Resting-State fMRI Data

**Figure 2:**
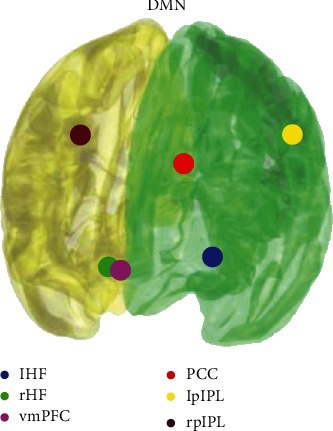
Seeds used for the rs-fMRI data analysis: default mode network. l/r a IPL = left/right anterior inferior parietal lobule; l/rHF = left/right hippocampal formation; vmPFC = ventromedial prefrontal cortex; PCC = posterior cingulate cortex; l/rIPL = left/right posterior inferior parietal lobule.

The mean rs-FC was assessed between the anode seed (rMFG) and the major DMN seeds (i.e., spheres with *r* = 6 mm) using six coordinates as described previously [2], see Figure 2. Representative mean seed signals were extracted, and correlation matrix was calculated for each subject. Pearson's correlation coefficients were converted using Fisher's *r*-to-*z* transformation to *z* values. The average connectivity between the anode seed and DMN seeds was calculated as the mean of *z* values for each seed pair. Because of the nonnormality of the data, Wilcoxon signed-rank tests were used to assess the tDCS-induced changes in the rs-FC. We further correlated differences of RT outcomes (RT post–RT baseline) with differences of *z* values of the connectivity changes (FC post–FC baseline) separately for real/sham condition.

## 3. Results

### 3.1. Demographic Data

The sample consisted of 31 right-handed young healthy volunteers. Altogether, data from four subjects were excluded: two participants did not finish the stimulation protocol due to health problems unrelated to the study protocol, and the data from two subjects were not fully recorded due to technical problems. The final dataset consists of 27 subjects, mean age 27 ± 4.1 years, 11 men and 16 women. A subsample of 22 subjects also underwent fMRI acquisition (mean age: 24.7 ± 4.6; 10 men and 12 women).

### 3.2. Behavioral Results

**Figure 3:**
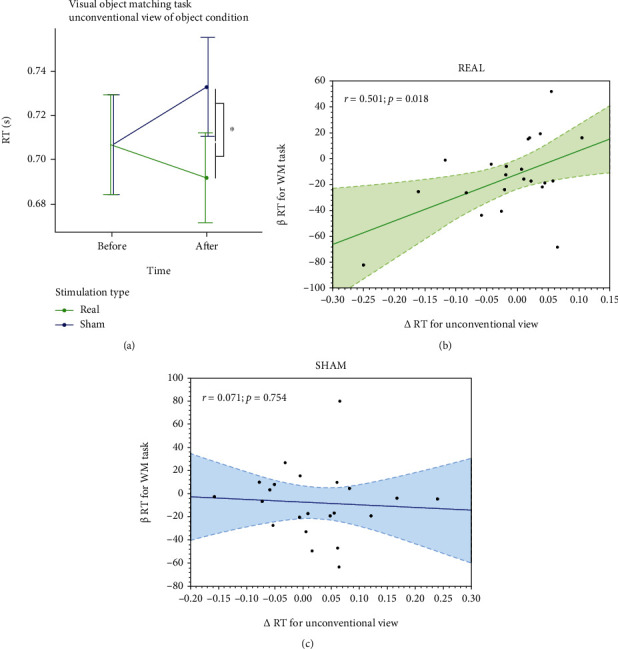
Behavioral results (a) offline VOMT (mean ± SE); (b) correlation of behavioral results online and offline tasks after tDCS (b) real and (c) sham ^∗^*p* < 0.05.

All subjects tolerated the stimulation well and mentioned only minor side effects (tingling, itching under the electrode) that faded away during the stimulation. As predicted, we observed a ceiling effect and low variability in correct responses for both the baseline VOMT subtask (median, IQR: 95%; 90-95%) and the baseline online WMT task (95%; 87-98%). Regarding RT changes, we found a significant difference between the real and sham stimulation conditions only for the VOMT subtask with more cognitively demanding unconventional views of objects, i.e., our subtask of interest (Δ RT_real_ = −0.015 s; Δ RT_sham_ = 0.026 s; *p* = 0.049), see Figure 3(a). As predicted, results for the whole VOMT (both subtasks with lower and higher cognitive load) were not significant (Δ RT_real_ = −0.007 s; Δ RT_sham_ = 0.02 s; *p* = 0.085). There was no significant difference in *β* RT for the online WMT task between active and sham conditions (see Supplementary Figure 1). Despite this negative effect, we observed that *β* RT for the online WMT correlated with *Δ* RT for the VOMT subtask with a higher cognitive load in the real but not in the sham tDCS condition (*r*_real_ = 0.501, *p* = 0.018; *r*_sham_ = 0.071, *p* = 0.754), see Figures 3(b) and 3(c).

### 3.3. fMRI Results

**Figure 4:**
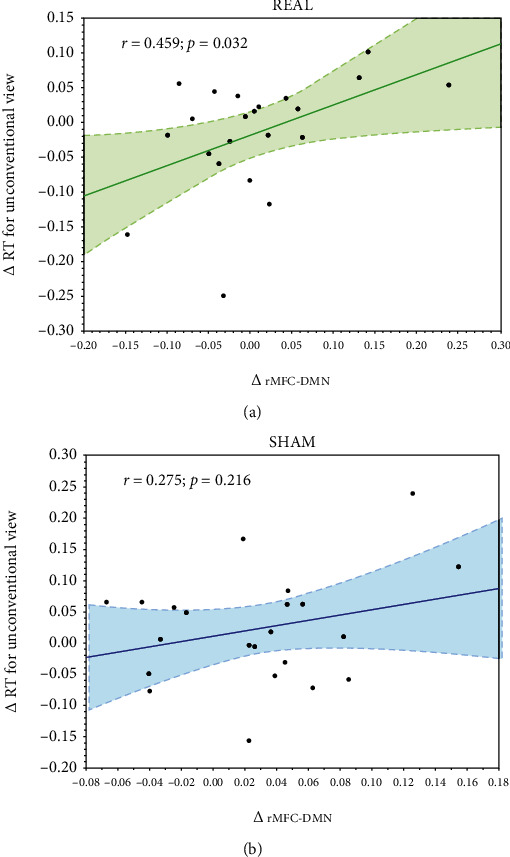
Correlation of behavioral results with rMFG–DMN rs-connectivity for (a) real and (b) sham conditions.

We found no differences between stimulation conditions (real and sham tDCS) in changes of the rMFG-DMN connectivity (*Δ* rMFG-DMN) (mean *z* values: Δ rMFG‐DMN_real_ = 0.006, Δ rMFG − DMN_sham_ = 0.028, *p* = 0.149); however, we observed a significant positive correlation between the *Δ* RT in the VOMT subtask with unconventional object view and *Δ* rMFG-DMN for the real tDCS (*r* = 0.459 and *p* = 0.032), see Figure 4. No such correlation was observed for the sham stimulation condition (*r* = 0.275; *p* = 0.216).

## 4. Discussion

In this study, we demonstrated that in young university-based healthy subjects, a single session of real tDCS over the right frontoparietal regions of the FPN was superior to sham tDCS in terms of affecting the cognitive speed of the cognitively more demanding subtask of the WMT (with unconventional views of objects). The impact of real tDCS on task accuracy could not be studied due to the ceiling performance of the task already at the baseline. Our results are consistent with previous studies in which only the cognitive speed of the WMT was enhanced after prefrontal tDCS stimulation in young healthy volunteers [37–41]. Two meta-analyses also indicated a favorable enhancement of reaction times with small but significant effect sizes in healthy populations as compared to accuracy improvement in patient samples with baseline WM deficits [42, 43]. However, null results of tDCS on cognition have also been reported [35, 44]. Among the factors that seem to influence the efficacy of tDCS in HY, the increased task load was shown to play an important role [31, 32, 37, 45].

Of note, the most common target of previous tDCS studies was the dorsolateral prefrontal cortex (DLPFC) [18, 42, 46]. Our anode was positioned in the anterior part of the right middle frontal gyrus, and the cathode was placed in the right posterior parietal cortex. This right-sided frontoparietal rather than bifrontal montage was based on our fMRI study with the same visual WMT [27]. In that study, deficient engagement of the right-sided FPN regions involved in working memory tasks and in the top-down control of visual processing [47–50] led to impaired task performance in patient groups as compared to healthy controls. In the current study, manipulating the excitability of the same right-sided FP regions by tDCS affected the processing speed of the WMT subtask with a higher cognitive load even in young healthy subjects. Taken together, using task fMRI for precise tDCS electrode placement may strengthen stimulation-induced cognitive aftereffects on that task. Future studies should explore whether such a specific modulation of the given WMT could also transfer to modulation of other cognitive tasks or even cognitive domains. We believe this might be possible particularly when domain-general regions of the FPN, known to be involved in top-down control of cognitive processing, are stimulated as in the current study.

We did not observe any significant modulation of the online WMT by tDCS. This finding is in line with the results of a meta-analysis by Hill et al. [43] that reported that anodal tDCS enhanced RTs of the offline WMTs in a healthy population; no significant effects were found for the online tasks. Despite insignificant results for the online WMT, we observed that the RT decreases of the online WMT in the real tDCS condition were associated with more pronounced cognitive speed changes of the offline VOMT subtask of interest. This finding may have clinical implications; future studies should examine whether the online cognitive speed modulation could predict offline aftereffects of multiple sessions of tDCS and thus help choose optimal candidates for long-term tDCS.

Our results may also be viewed in a neurocognitive theorem of object-tool processing and recognition. The right FPN (the main target of our tDCS stimulation) is actively implicated in high-level executive functions [2]. Recent evidence indicates its specific role in object and tool recognition [47, 48, 50–52] through the concept of “action reappraisal.” Action reappraisal is a multidimensional cognitive process combining multiple sources of information (e.g., semantic knowledge, mechanical knowledge, and sensorimotor knowledge) processed in a recursive semantic-to-mechanical-to-motor “cascade” [53], subserved by a dynamic interaction of complex brain networks (particularly the interplay between the frontoparietal and occipitotemporal networks), thus providing a generalizable and in everyday context usable object representations [51, 52, 54–56]. A series of behavioral studies employing eye tracking provided evidence that higher-level information is activated earlier than lower-level perceptual information and can affect a visuoattentional pattern in which the objects are processed, although the magnitude of top-down processes is modulated by context and expectations [54–56]. The action reappraisal concept, as part of the reasoning-based framework [53], provides an alternative to the well-established embodied-cognition approach which suggests that object knowledge is constituted by information inscribed within the motor and sensory systems thus stressing the automatic lower-level processing of information [57]. In the current study, we modified the speed of object and tool recognition in the offline VOMT task by targeting the FPN (and the right MFG in particular) by tDCS. We thus provide the first empirical “causal” evidence for the action reappraisal mechanism of object and tool processing. Interestingly, this effect seemed to be modality specific as no significant effects were found for the online WMT comprising faces and scenes stimuli. Notably, other explanations for the modality-specific aftereffects are possible as different effects of offline and online stimulation on various WMTs have been observed [58].

The tDCS-induced rs-FC changes between the anode seed (engaged in the task-positive FPN) and the DMN seeds were not significantly different between the real and sham stimulation conditions. However, our behavioral results correlated with the real tDCS-induced rs-FC changes between the frontal hub of the FPN (our anode seed) and the DMN such that the VOMT subtask speeding was linked to between-network connectivity decreases. Previous studies demonstrated that many cognitive functions, including working memory, are processed within large-scale brain networks and are dependent on their dynamic cross-talk. For example, decreases of functional connectivity between the task-positive FPN and DMN were linked to better performance of a WMT that was further strengthened by online frontal tDCS [59]. Conversely, an increase of rs-FC between the FPN and the DMN was associated with negative cognitive outcomes in healthy subjects and in patient groups [1, 60, 61]. Such an increase of the between-network rs-FC could be related to a higher propensity to mind wandering and lapses of attention [62], which may in turn lead to cognitive slowing [63–65]. Here, we show for the first time that manipulating right-sided FPN by tDCS during concurrent cognitive training may modulate the speed of top-down processing of an offline visual WMT (i.e., measured after the intervention and compared to baseline performance) that is linked to the offline rs-FC decreases between task-positive and task-negative brain networks. Similar brain-behavior associations were recently shown after concurrent WMT training with frontal tDCS, such that decreases of activity in the anterior DMN node correlated with faster responses as measured by offline task-fMRI [66]. However, the results of rs-FC and task-related activations cannot be directly compared.

## 5. Conclusion

We demonstrated cognitive speed modulation of the offline WMT in young healthy subjects that was induced by a single session tDCS coupled with the online cognitive training as compared to online cognitive training alone (coupled with sham tDCS). We provide the first empirical evidence for the action reappraisal mechanism of object and tool processing. The behavioral tDCS-induced changes were correlated with the magnitude of the FPN-DMN cross-talk changes after the real stimulation condition. Therefore, the between-network rs-FC measures may be used to monitor tDCS-induced cognitive aftereffects.

## Data Availability

The datasets used for supporting the findings in this study are available from the corresponding author on reasonable request.

## Supplementary Materials

Supplementary Materials Behavioral results of the online WMT task might be seen in Supplementary Figure 1.
