# Supplementary material for: Modulation of Working Memory and Resting-State fMRI by tDCS of the Right Frontoparietal Network
Source: Neural Plast. 2021 Jul 26;2021:5594305. doi: 10.1155/2021/5594305 (PMC8328716; doi:10.1155/2021/5594305)
Supplement: Supplementary Materials — Behavioral results of the online WMT task might be seen in Supplementary Figure 1. [file 5594305.f1.pdf]

Supplementary material

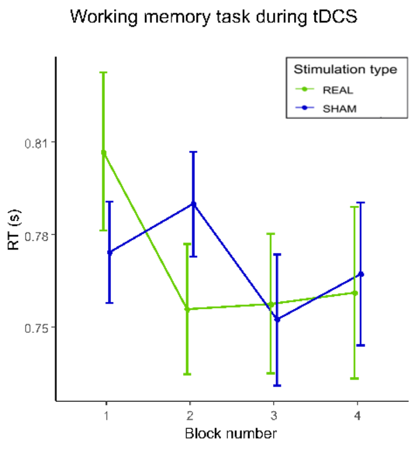

Figure 1: Behavioural results for online WMT (mean ± SE)
